# Evaluations and Mechanistic Interrogation of Natural Products Isolated From *Paeonia suffruticosa* for the Treatment of Inflammatory Bowel Disease

**DOI:** 10.3389/fphar.2021.696158

**Published:** 2021-12-06

**Authors:** Kun-Chang Wu, Der-Yen Lee, Jeh-Ting Hsu, Chi-Fang Cheng, Joung-Liang Lan, Shao-Chih Chiu, Der-Yang Cho, Jye-Lin Hsu

**Affiliations:** ^1^ School of Pharmacy, College of Pharmacy, China Medical University, Taichung, Taiwan; ^2^ Graduate Institute of Integrated Medicine, China Medical University, Taichung, Taiwan; ^3^ Department of Information Management, Hsing Wu University, New Taipei, Taiwan; ^4^ Graduate Institute of Biomedical Sciences, China Medical University, Taichung, Taiwan; ^5^ Division of Rheumatology and Immunology and Department of Internal Medicine, China Medical University Hospital, Taichung, Taiwan; ^6^ College of Medicine, China Medical University, Taichung, Taiwan; ^7^ Drug Development Center, China Medical University, Taichung, Taiwan; ^8^ Translational Cell Therapy Center, Department of Medical Research, China Medical University Hospital, Taichung, Taiwan; ^9^ Department of Neurosurgery, China Medical University Hospital, Taichung, Taiwan

**Keywords:** anti-inflammation, traditional Chinese medicine, moutan radicis cortex, inflammatory bowel disease, mu dan pi, Paeonia suffruticosa

## Abstract

Mu Dan Pi (MDP), a traditional Chinese medicine derived from the root bark of *Paeonia suffruticosa* Andrews, is used to treat autoimmune diseases due to its anti-inflammatory properties. However, the impact of MDP on inflammatory bowel disease (IBD) and its principal active compounds that contribute to the anti-inflammatory properties are uncertain. Thus, this study systemically evaluated the anti-inflammatory effects of fractionated MDP, which has therapeutic potential for IBD. MDP fractions were prepared by multistep fractionation, among which the ethyl acetate-fraction MDP5 exhibited the highest potency, with anti-inflammatory activity screened by the Toll-like receptor (TLR)-2 agonist, Pam3CSK4, in a cell-based model. MDP5 (at 50 μg/ml, *p* < 0.001) significantly inhibited nuclear factor kappa-B (NF-κB) reporters triggered by Pam3CSK4, without significant cell toxicity. Moreover, MDP5 (at 10 μg/ml) alleviated proinflammatory signaling triggered by Pam3CSK4 in a dose-dependent manner and reduced downstream IL-6 and TNF-α production (*p* < 0.001) in primary macrophages. MDP5 also mitigated weight loss, clinical inflammation, colonic infiltration of immune cells and cytokine production in a murine colitis model. Index compounds including paeoniflorin derivatives (ranging from 0.1 to 3.4%), gallic acid (1.8%), and 1,2,3,4,6-penta-*O-*galloyl-β-*D*-glucose (1.1%) in MDP5 fractions were identified by LC-MS/MS and could be used as anti-inflammatory markers for MDP preparation. Collectively, these data suggest that MDP5 is a promising treatment for IBD patients.

## Introduction

Inflammatory bowel diseases (IBDs) are inflammatory disorders of the gastrointestinal tract that affect millions of individuals worldwide, at increasingly higher rates (Ng et al., 2018). The two major disorders of IBD, Crohn’s disease (CD) and ulcerative colitis (UC), are characterized by both acute and chronic inflammation of the intestine with multifactorial etiology. Aminosalicylates are typically the first medications to be used in IBD (Colombel et al., 2010). If the patient’s condition fails to respond to the aminosalicylate therapy, the second step is often a corticosteroid, which rapidly relieves symptoms, but could have adverse effects (van den Brande et al., 2006; Colombel et al., 2010; Laharie et al., 2018). Biologic agents are very effective in certain patients but one-third are resistant to anti-tumor necrosis factor-alpha (TNF-α) therapies, while another third experience loss of therapeutic efficacy with anti-TNF-α treatment over time (Gole and Potocnik, 2019). Moreover, biologic agents are pricey. Thus, drug development needs to have alternative strategies for IBD.

Traditional Chinese medicine (TCM) has long been used in Asian countries to treat various autoimmune and inflammatory diseases (Weng et al., 2016). In Taiwan, recent reports have recorded TCM use in therapeutic regimens by 37% of patients with IBD (Chen et al., 2008), 70% of patients with psoriasis (Weng et al., 2016) and 27% of patients with rheumatoid arthritis (Huang et al., 2015). Of all TCMs, Mu Dan Pi (MDP) is one of the most commonly prescribed single TCM herbs for the treatment of inflammatory symptoms and psoriasis (Weng et al., 2016). MDP, also known as Moutan Radicis Cortex, is derived from the root bark of *Paeonia suffruticosa* Andrews (Genus: *Paeonia*; Fam: *Paeoniaceae*) (Zong et al., 2018). Its bioactive components exhibit antioxidant, antimicrobial and anticancer properties (Wang et al., 2017; Li et al., 2018; Liu et al., 2018; Tsang et al., 2018; Yoo et al., 2018) and are widely used in the treatment of diabetes, neurological disorders, cardiovascular diseases and cancer (Wang et al., 2017; Liu et al., 2018; Tu et al., 2019). However, it remains unclear as to which component most effectively provides the anti-inflammatory activities of MDP. Furthermore, the diarrhea associated with MDP (due to contaminated impurities or its derived components) reduces the option of long-term use. It is therefore necessary to remove the impurities and identify the possible active components that can be used for treating IBD.

The pathogenesis of IBD involves innate immune responses to pathogen-associated molecular patterns residing in pathogens, such as lipopolysaccharide (LPS), peptidoglycan, lipoteichoic acid, and lipoproteins. These molecular patterns can activate pattern recognition receptors (PRRs) and downstream proinflammatory signaling. The early detection of microbes by PRRs, such as toll-like receptors (TLRs), is critical for initiating the innate immune responses required to provide protection for the host. Among PRRs, TLR2 plays a crucial role in detecting a family of cell wall components in not only Gram-positive and Gram-negative bacteria (Oliveira-Nascimento et al., 2012), but also fungi and viruses (Henrick et al., 2015). These ligands activate TLR2 primarily by the TLR2-IRAK4 (interleukin-1 receptor-associated kinase 4)-nuclear factor kappa-light-chain-enhancer of activated B cells (NF-κB) pathway, which stimulates the transcription of various cytokines such as interleukin-1 (IL)-1β, IL-6 and TNF-α, to counteract infection (Mogensen, 2009). In addition to their anti-infective roles, high levels of these proinflammatory cytokines are found in inflammatory conditions and in IBD in particular. This study therefore screened various anti-inflammatory fractions from MDP in an attempt to identify the most effective fraction and its derived small molecules that mediate TLR2-IRAK4-NF-κB signaling. Applying the TLR agonists to activate macrophages may further dissect the TLR signaling pathways and determine whether MDP plays a role in inhibiting inflammatory pathways triggered by bacteria.

We have previously reported on the anti-inflammatory activity of our aqueous extraction of MDP (Chen et al., 2020). We also compared this MDP aqueous extract with all reported anti-inflammatory compounds identified in MDP (Chen et al., 2020). However, the impact of MDP on IBD and its principal active compounds that contribute to the anti-inflammatory properties remain uncertain. Thus, we attempted to isolate and evaluate the fraction of MDP with the maximum therapeutic effect and minimal adverse effects for IBD patients and consequently identify individual compounds in MDP5 by LC-MS/MS, hoping to identify some for use as anti-inflammatory markers for MDP preparation.

## Materials and Methods

### Preparation of MDP Fractions

**FIGURE 1:**
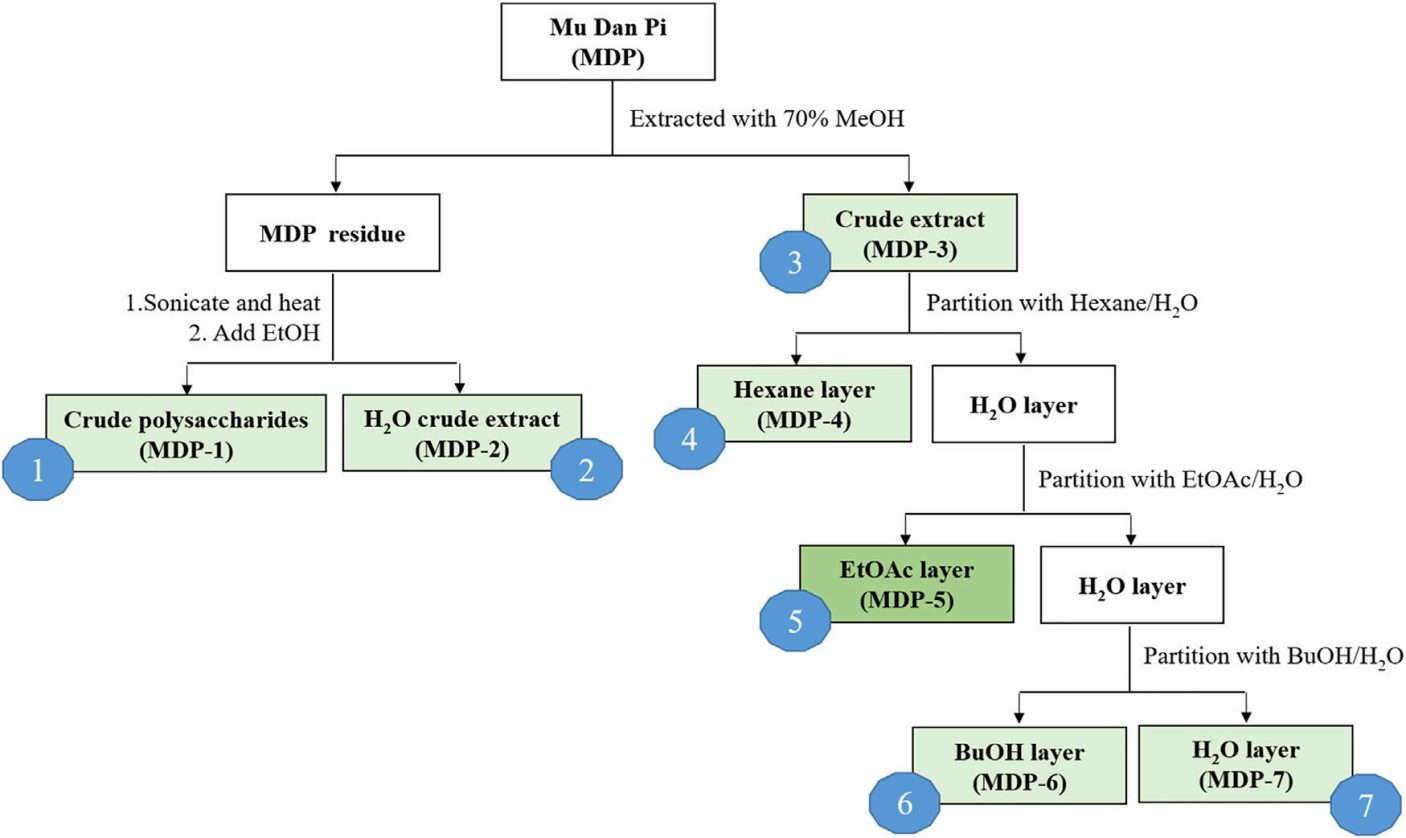
Flow chart of the preparation procedures for MDP fractions.

The dried root bark of *Paeonia suffruticosa* Andrews was purchased from a local herbal medicine store in Taichung, Taiwan, and authenticated by Dr. Kun-Chang Wu. Solvents used for extraction and partition activities, including *n*-hexane, ethyl acetate (EtOAc), acetone, *n*-butanol (*n*-BuOH), methanol (MeOH) and ethanol (EtOH), were all of ACS grade. The extraction and partition procedures are illustrated in Figure 1. Crushed MDP (100.24 g) was extracted with 70% MeOH (1 L, four times). The MDP residue was dried and subsequently used to produce crude polysaccharides (MDP1) and hot water crude extract (MDP2). The MDP residue (57.58 g) was subjected to ultrasonication and boiled four times in 20-fold distilled water at 85°C for 1.5 h. A part of the hot water extract was then evaporated under reduced pressure to reduce the volume, and a 4-fold volume of 95% ethanol was added to precipitate MDP1. The remaining hot water extract was dried using a rotary evaporator to obtain MDP2. The 70% MeOH extract solution was filtered and the filtrate was concentrated by evaporation under reduced pressure to obtain a 70% MeOH crude extract (MDP3, 32.85 g, yield rate: 32.77%). A portion of the MDP3 (20 g) was suspended in distilled water and the aqueous suspension was sequentially partitioned with *n*-hexane, EtOAc, and *n*-BuOH. The *n*-hexane-soluble (MDP4), EtOAc-soluble (MDP5), *n*-BuOH-soluble (MDP6) and H_2_O-soluble (MDP7) layers were obtained for subsequent pharmacological experiments.

### Cell Culture Conditions and Reporter Assays

THP-1 cells and reporter cells were cultured according to a previously described protocol (Chen et al., 2020). Bone marrow-derived macrophages (BMDMs) were isolated from mice and differentiated for 5–6 days, following standard procedures (Hsu et al., 2015). THP-1 cells or BMDMs were treated with MDP fractions at the indicated concentrations for 6 h. The cells were then stimulated with 0.2 μg/ml Pam3CSK4 (tlrl-pms, InvivoGen) or 0.1 ng/ml LPS (SI-L8274, Sigma-Aldrich). After stimulation for 14–16 h, NF-κB activity was quantified by measuring the levels of alkaline phosphatase in the culture supernatant. Changes from baseline were calculated as -fold changes and normalized against unstimulated control cells.

### Cell Viability Assays

The viability of THP-1 cells was analyzed using the MTS assay. THP-1 cells were pretreated with MDP fractions with or without ligand stimulation. After stimulation for the indicated times, the cells were washed twice with fresh medium. Cell viability was analyzed using an MTS assay kit (ab197010, Abcam) that determined the amount of 490 nm absorbance after 2 h of incubation. The percentage of cell death was calculated by normalization with cells that were either untreated or treated with the ligand only.

### Animals and the Dextran Sodium Sulfate-Induced Colitis Model

Male C57BL/6 mice aged 8–12 weeks were kept in a pathogen-free environment during the experiments. DSS-induced colitis was induced by the administration of 3% DSS (36,000–50,000 molecular weight; 0216011080/B9, MP Biomedicals) in the drinking water from day 0 to day 5, followed by 6 days of normal water, as previously reported (Zaki et al., 2010). In the drug treatment experiment, mice were randomly assigned to one of two groups; the vehicle control or treatment group. In the treatment group, mice were orally gavaged daily with MDP5 (20 mg/kg) or mesalazine (20 mg/kg, FM2511, Carbosynth), starting from day 0. Both drugs were dissolved in DMSO with vehicle (0.5% methyl cellulose and 0.1% tween-80). Body weights and clinical scores were monitored daily. Scoring for stool consistency and occult blood was done as described previously (Demon et al., 2014). Briefly, stool scores were determined as follows: 0 = well-formed pellets, 1 = semi-formed stools that did not adhere to the anus, 2 = semi-formed stools that adhered to the anus, 3 = liquid stools that adhered to the anus. Bleeding scores were determined as follows: 0 = no blood as according to Hemoccult SENSA (64,152, Beckman Coulter), 1 = positive Hemoccult SENSA, 2 = visible blood traces in stool, 3 = rectal bleeding. Stool and bleeding scores were averaged to calculate the clinical score. On day 11, the colon samples were collected, measured and photographed. All animal care and experimental methods were approved by the Institutional Animal Care and Use Committee of China Medical University, Taichung, Taiwan and were performed in line with the Guideline for the Care and Use of Laboratory Animals and approved by Taiwan’s government organization, Council of Agriculture, Executive Yuan.

### Immunoblotting Analysis

BMDMs were treated with MDP fractions at the indicated concentrations for 2 h, then stimulated with 0.2 μg/ml Pam3CSK4 for 1 h. Cell lysate was quantified, analyzed by SDS-PAGE, and blotted with antibodies as follows: glyceraldehyde 3-phosphate dehydrogenase and other antibodies were provided by Cell Signaling, including p-transforming growth factor-β activated kinase 1 (TAK1, 4,536), TAK1 (5,206), p-IRAK4 (11,927), IRAK4 (4,363), p-IκB kinase (IKK) α/β (2,697), IKKα (61,294), p-inhibitor of nuclear factor kappa B (IκB)α (2,859), and IκBα (4,814).

### Enzyme-Linked Immunosorbent Assay and Immunochemistry Staining

ELISA kits for detecting mouse IL-1β (88-7,013), IL-6 (88-7,064) and TNF-α (88-7,324) were obtained from eBioscience. Cytokine measurements were performed according to the respective manuals provided by eBioscience. Antibodies of CD3 (A0452) and F4/80 (MCA497G) were purchased from DAKO and Bio-Rad. Immunochemistry staining was performed according to the respective manuals provided by the manufacturers (Kafil and Sharifi, 2021).

### LC-ESI-MS

MDP5 stock was diluted in ultrapure water to the final concentration of 100 ppm. The liquid chromatography-electrospray ionization-mass spectrometry (LC-ESI-MS) system consisted of an ultra-performance liquid chromatography (UPLC) system (ACQUITY UPLC I-Class, Waters) using a 4 kDa quadrupole time-of-flight (TOF) mass spectrometer (Waters VION, Waters) as the ESI/atmospheric pressure chemical ionization (APCI) source. The flow rate was set at 0.2 ml/min with the column temperature at 35°C. Separation was performed with reversed-phase liquid chromatography (RPLC) on a BEH C18 column (2.1 × 100 mm, Walters) with a 7.5 μl sample injection. The elution started from 99% mobile phase A (ultrapure water +0.1% formic acid) and 99% mobile phase B (100% methanol +0.1% formic acid), was held at 1% B for 0.5 min, raised to 90% B over 5.5 min, held at 90% B for 1 min, then lowered to 1% B over 1 min. The column was equilibrated by pumping 1% B for 4 min. LC-ESI-MS chromatograms were acquired under ESI mode using the following conditions: a capillary voltage of 2.5 kV, a source temperature of 100°C, a desolvation temperature of 250°C, maintenance of cone gas at 10 L/h and desolvation gas at 600 L/h, and acquisition by MS^E^ mode with a range of m/z 100–1,000 and 0.5 s scan time. The compound library was established with reference to a review article (Wang et al., 2017) and applied to identify candidates in the acquired data, which were processed by UNIFI software (Waters) with illustrated chromatograms and summarized in an integrated area of signals.

### Statistical Analysis

Results are presented as the mean ± S.E.M. in mouse model experiments and as the mean ± standard deviation (S.D.) in experiments using primary macrophages or THP-1 cells. Data were analyzed using the Student’s *t*-test (two-tailed) or two-way ANOVA with the multiple comparison test. A *p*-value of <0.05 was considered significant.

## Results

### Comparative Evaluation of the Anti-Inflammatory Activity of Fractionated MDP on the NF-κB Reporter

**FIGURE 2:**
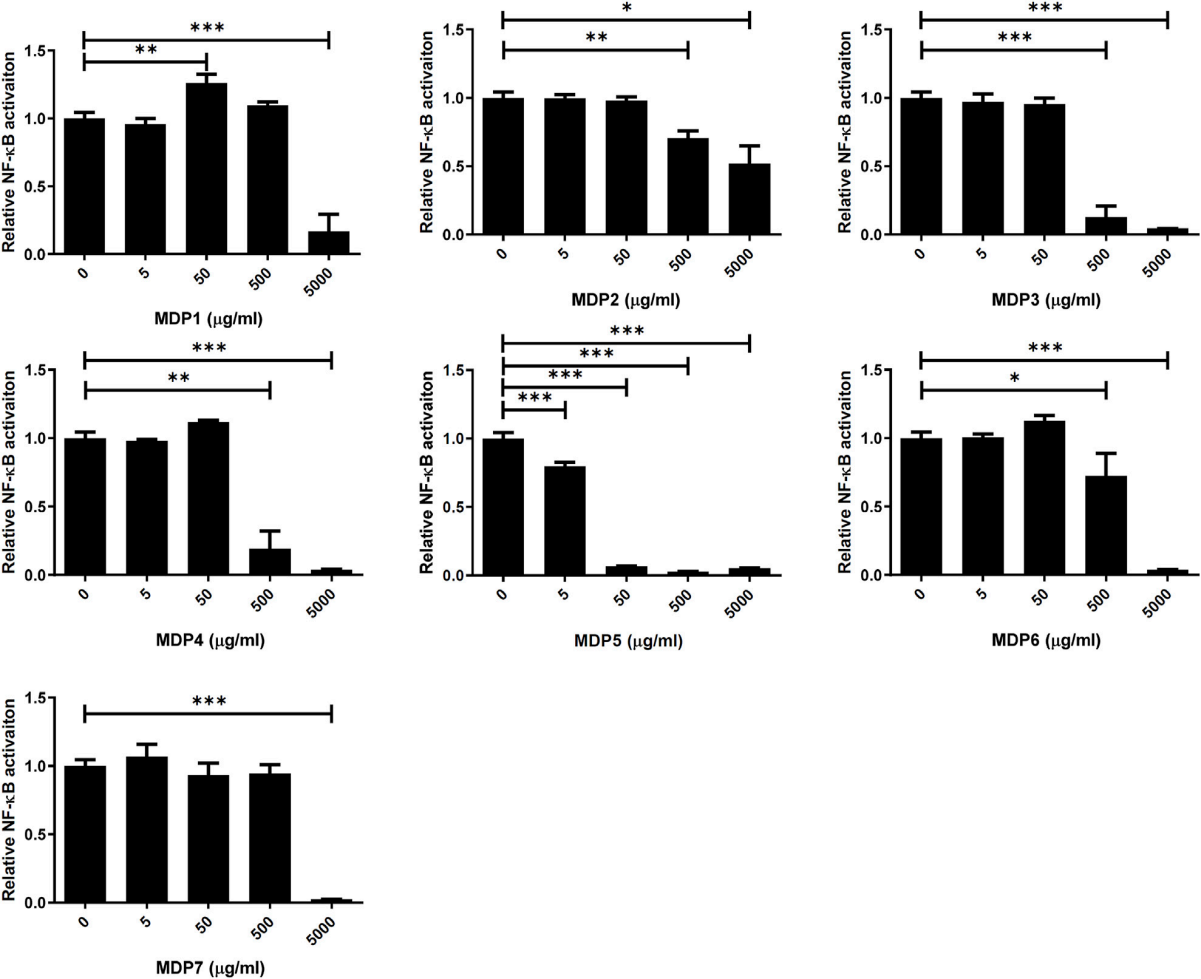
Screening of inhibitory effects of MDP fractions on the NF-kB reporter. THP-1 cells were treated with different concentrations of individual MDP fractions (0, 5, 50, 500, or 5,000 ug/ml) and stimulated with the TLR2 ligand, Pam3CSK4. After stimulation for 24 h, promoter activities were measured. Data are the mean ± S.D. (*n* = 3–5). **p < 0.01, ***p < 0.001. Data were analyzed using the Student’s *t*-test (two-tailed).

**FIGURE 3:**
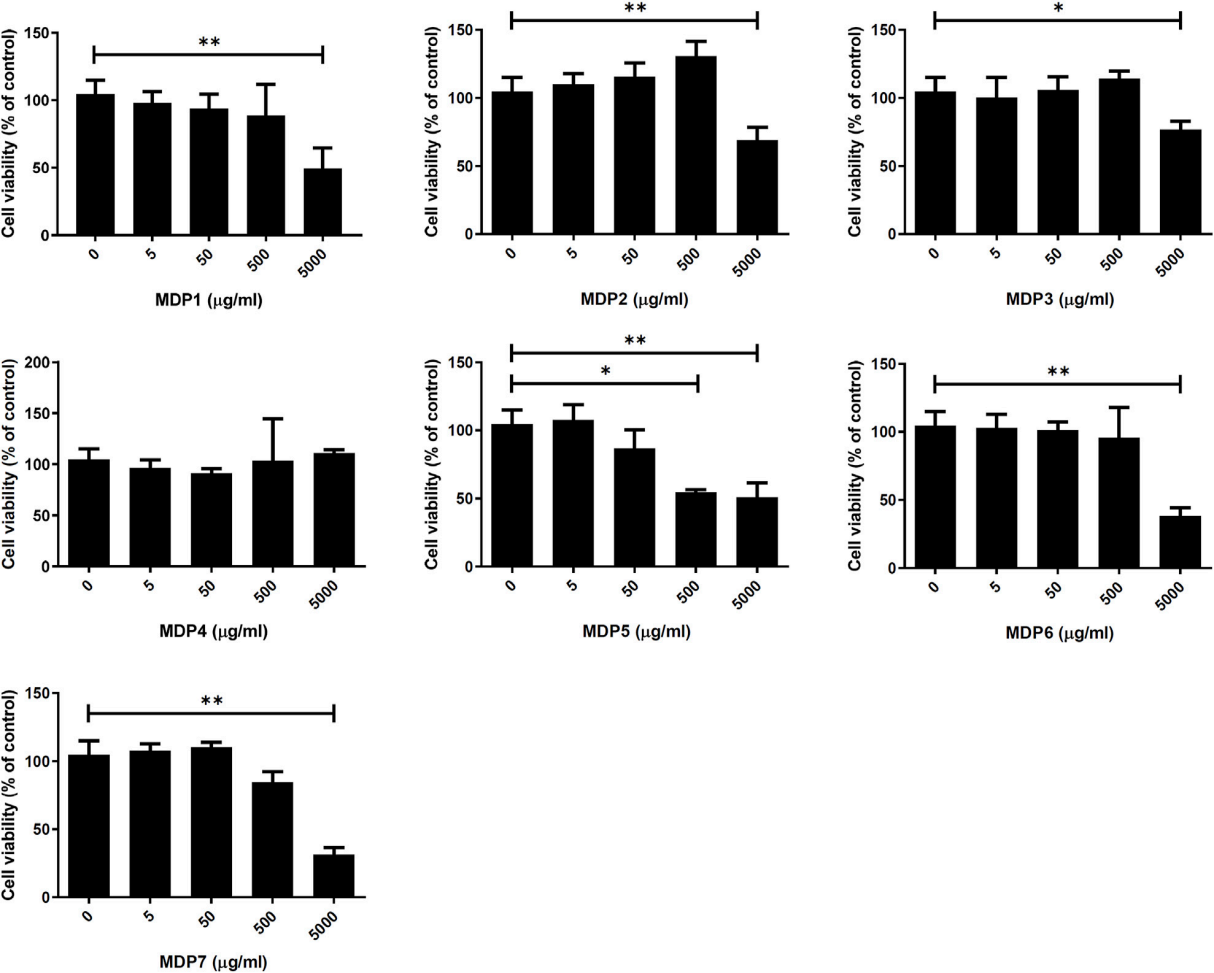
Cell toxicity of MDP fractions. THP-1 cells were treated with different concentrations of individual MDP fractions and stimulated with Pam3CSK4. After 24 h of stimulation, THP-1 cell viability was determined by the MTS assay. Data are the mean ± S.D. (*n* = 3–5). **p < 0.01, ***p < 0.001. Data were analyzed using the Student’s *t*-test (two-tailed).

We first processed MDP using serial extraction and partition, in order to isolate the fraction with the maximum therapeutic efficacy and minimal adverse effects. Crushed MDP was extracted with 70% methanol and subsequently partitioned with *n*-hexane, ethyl acetate and *n*-butanol, then named as MDP1 through MDP7, according to the order of partitioning (Figure 1). Next, we screened MDP fractions using cell-based reporter assays to identify the most effective fractions contributing to the anti-inflammatory effects of MDP. The monocytic cell line THP-1, which stably expresses NF-κB reporter constructs, was used as the reporter cells as previously described (Chen et al., 2020). NF-κB reporter activity, enabling verification of the inflammatory response to the TLR2 agonist, Pam3CSK4, was used to measure inhibitory activity of each MDP fraction (Figure 2). Among all fractions, MDP5 displayed the highest level of inhibition against promoter activity, with the lowest IC_50_ (∼50 μ g/ml) (Figure 2). Although some deaths occurred amongst activated THP-1 cells subjected to a high concentration of MDP5 (500 μ g/ml) (Figure 3), no cell deaths were observed at 50 μ g/ml with or without TLR-2 activation (Figure 3 and Supplementary Figure S1), suggesting that MDP5 at its effective concentration has very low cell toxicity. In contrast, MDP1 at ∼50 μ g/ml enhanced NF-κB reporter activity (Figure 2), suggesting that there may be an inflammatory-stimulated component in this fraction. All other fractions, including MDP2, MDP3 and MDP4, displayed moderate inhibitory activity, which reduced reporter activity at a relatively high concentration (∼500 μ g/ml).

### MDP5 Demonstrated the Most Significant Inhibitory Activity Against Inflammatory Signaling and Downstream Inflammatory Cytokine Production Triggered by a TLR-2 Agonist

**FIGURE 4:**
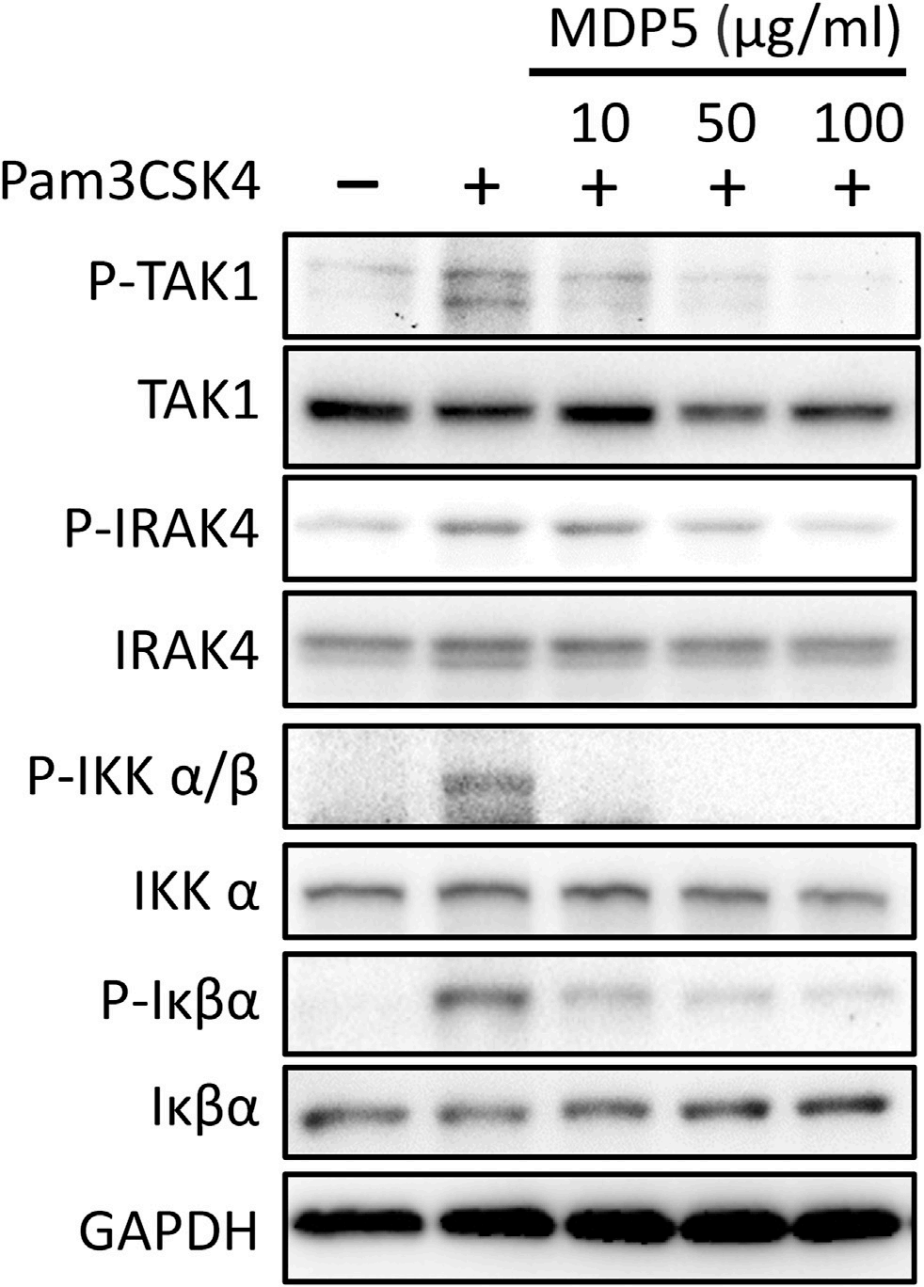
MDP5 prevented NF-κB signaling activation in BMDMs. BMDMs were pretreated with MDP5 (10–100 ug/ml)) for 2 h then stimulated with Pam3CSK4 for 1 h. Activation of signaling in BMDMs is shown by immunoblots using the indicated antibodies.

Amongst the fractions tested, MDP5 was the most effective fraction for reducing NF-κB reporter activation in THP-1 cells. Thus, we sought to verify the activity of MDP5 in TLR2-mediated signaling that triggers the activation of IRAK4, TAK1 and downstream NF-κB. Immunoblotting analysis revealed that the phosphorylated forms of IRAK4, TAK1, IKKα/β and Iκβα were all reduced in BMDMs treated with MDP5, in a dose-dependent manner (Figure 4), suggesting that the signaling of TLR2 was alleviated by MDP5.

**FIGURE 5:**
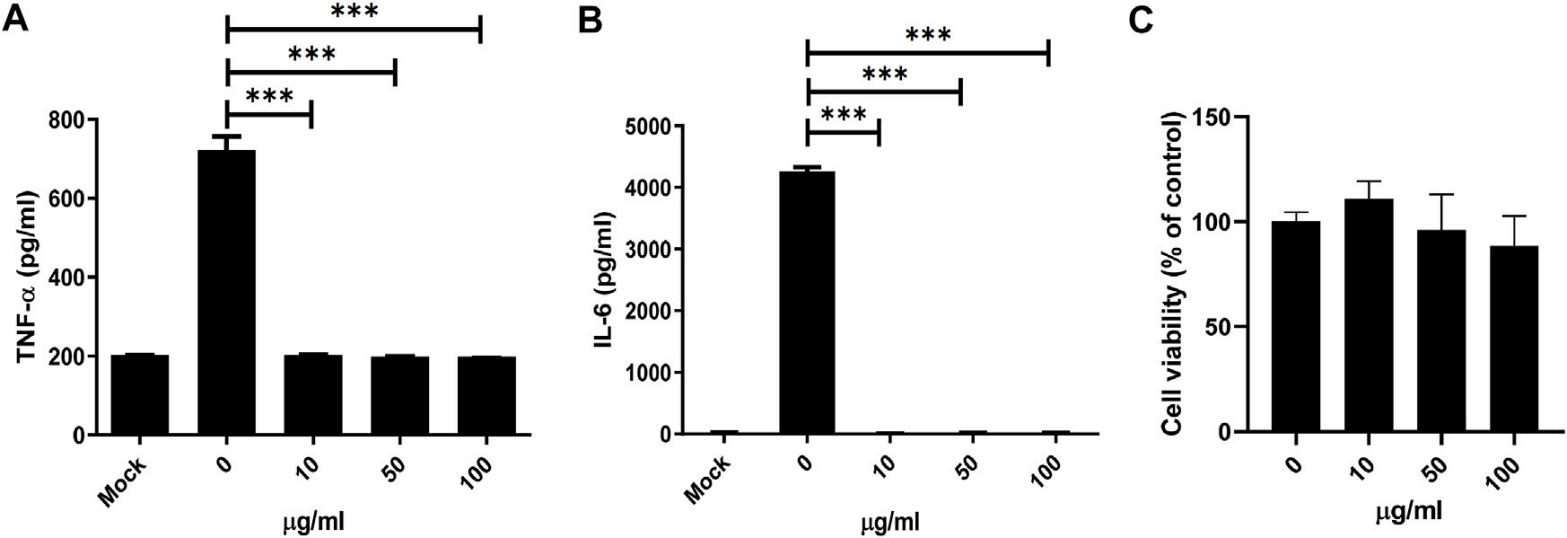
MDP5 inhibited NF-κB downstream cytokine production in BMDMs. BMDMs were pretreated with MDP5 (10–100 ug/ml) for 6 h then stimulated with Pam3CSK4 for cytokine production. Mock was shown by using BMDMs without Pam3CSK4. IL-6 **(A)** and TNF-α **(B)** production and viability **(C)** of BMDMs was measured after 12 h of Pam3CSK4-induced stimulation. Data are the mean ± S.D. (*n* = 5). **p < 0.01, ***p < 0.001. Data were analyzed using the Student’s *t*-test (two-tailed).

Consequently, inflammatory cytokines downstream of NF-κB were measured. Secretion of IL-6 and TNF-α from BMDMs was significantly reduced under MDP5 treatment at ∼50 µM (Figures 5A,B), with negligible levels of cell death (Figure 5C), suggesting that MDP5 can alleviate NF-κB reporter activation and inflammatory cytokine production.

### MDP5 Reduces Inflammation in the DSS-Induced Colitis Mouse Model

**FIGURE 6:**
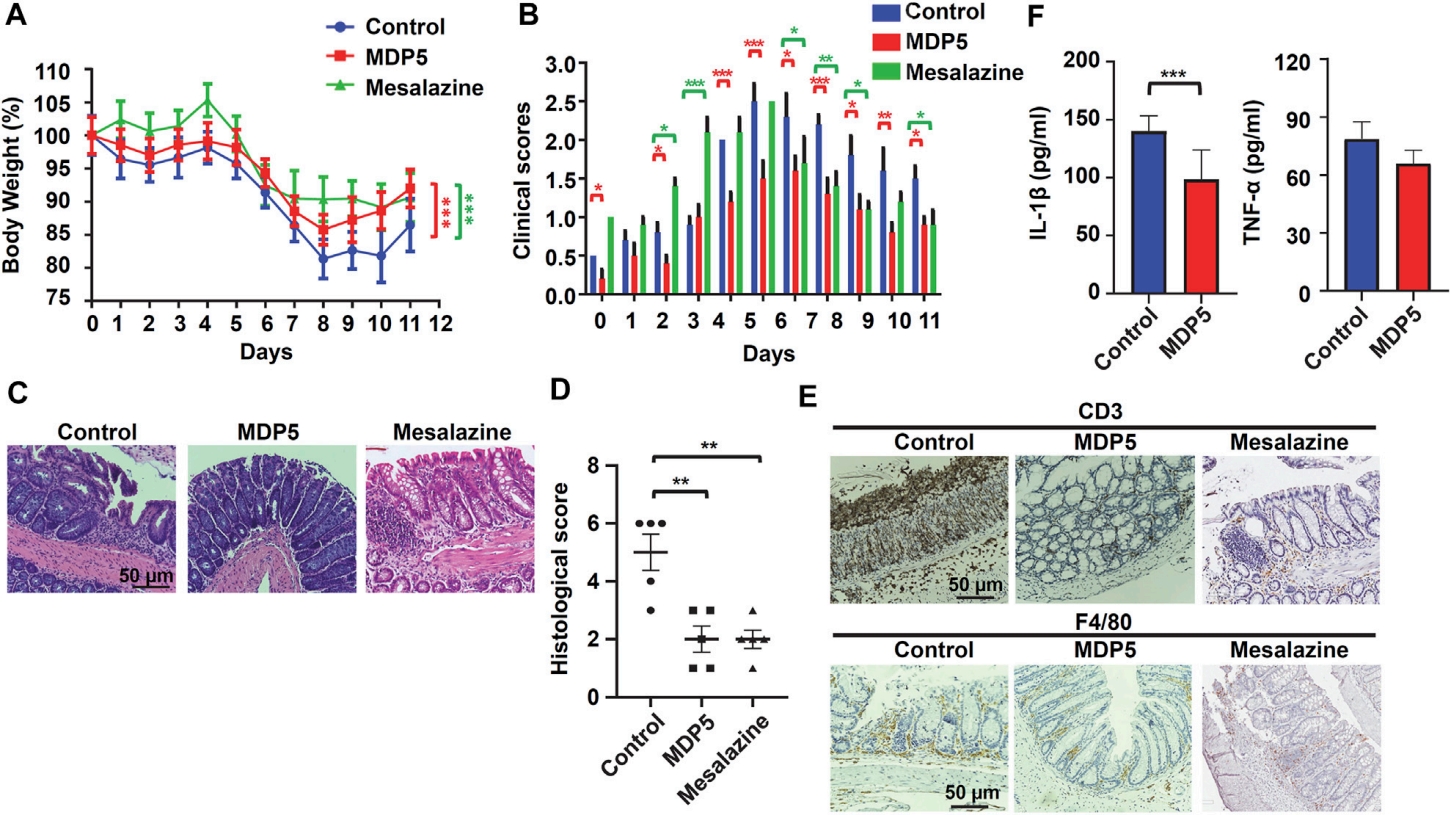
MDP5 alleviated DSS-induced colitis in mice. Mice were orally gavaged with MDP5 (20 mg/kg) or mesalazine (20 mg/kg) and colitis was induced with 3% DSS for 5 days (*n* = 5 per group). **(A)** Percentage of body weight. **(B)** Clinical scores. **(C,E)** Images of H&E-stained **(C)** and immunohistochemistry-stained **(E)** colon sections from mice scarified on day 11. Intestinal inflammation, infiltration of immune cells and quantities of cytokines were assessed by histological scoring **(D)**, immunochemistry staining **(E)**, and ELISA **(F)**, as described in the Methods section. Data were analyzed using the Student’s *t*-test (two-tailed) **(A,D,F)** or two-way ANOVA with the multiple comparison test **(B)**.*p < 0.05, **p < 0.01.

Given that MDP5 has strong anti-inflammatory effects, we therefore examined whether the fraction can be applied in the treatment of IBD in a colitis mouse model. Colitis was induced by adding 3% DSS to the drinking water for 5 days, followed by normal water for 6 days. In the treatment group, MDP5 and mesalazine (5-aminosalicylic acid) were provided daily by oral gavage. Mesalazine, a common clinical drug for IBD, was used as an effective treatment control. Body weight percentages and clinical scores were examined daily. During days 8 through 11, MDP5 mitigated the phenotypes of DSS-induced colitis, as evidenced by smaller amounts of body weight loss (Figure 6A) and lower clinical inflammation scores (Figure 6B), compared with placebo control mice. Pathological analysis of histological score by hematoxylin and eosin staining of colonic sections demonstrated less colonic inflammation and lower amounts of submucosa damage in the MDP5 treatment group (Figures 6C,D). Fewer tissue-infiltrated immune cells were found in the MDP-treated mice compared with placebo controls in immunochemistry staining of anti-CD3 antibodies and anti-F4/80 (activated macrophages) (Figure 6E). Examination of key inflammatory cytokines, IL-1β and TNF-α, revealed a significant reduction in IL-1β levels in the MDP5 treatment group (Figure 6F). Although clinical scores were higher in mesalazine-treated mice than in those treated with MD5 (Figure 6B), mesalazine showed similar effects to MDP5 in the mitigation of DSS-induced colitis (Figures 6A–D) and reductions in colonic inflammation and immune cell infiltration (Figure 6E). When we characterized the safety profile of MDP5 in a mouse model (Supplementary Figure S2), there was no evidence of body weight loss or changes in clinical inflammation scores in mice fed oral MDP5 daily for 11 days (Supplementary Figure S2A). Indicators of liver function, aspartate aminotransferase (AST/GOT) and alanine aminotransferase (ALT/GPT), and also kidney function, blood urea nitrogen (BUN) and creatinine (CRE), did not differ between MDP5-treated mice and controls on day 11 (Supplementary Figure S2B), suggesting that MDP5 can not only effectively mitigate weight loss and clinical inflammation in a murine colitis model, but also work safely. All findings suggested that MDP5 is highly effective at resolving immune cell activation and infiltration in the gut, reducing colitis severity in a murine model.

### The Chemoprofile and Index Compounds of MDP5

**FIGURE 7:**
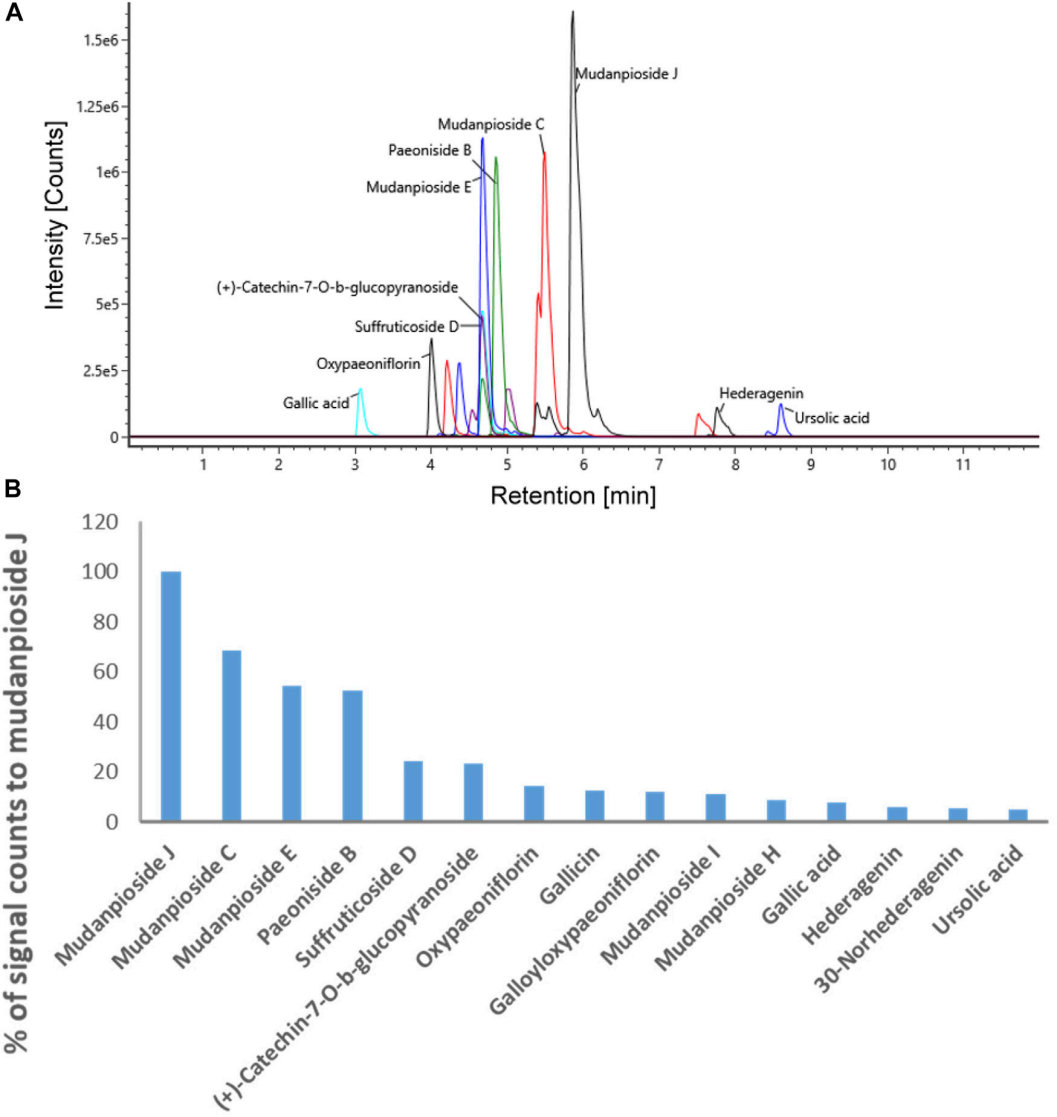
The MDP5 chemoprofile. **(A)** The extracted ion chromatograms (theoretical m/z ±16.8 ppm) with signal responses among the top 15 of all identified compound candidates. **(B)** The relative levels of signal responses amongst the compound candidates.

In order to use MDP5 in clinics, it is crucial to fingerprint MDP5 to ensure consistent therapeutic efficacy between different batches. We therefore performed a LC-ESI-MS analysis to identify index compounds with known chemical structures in MDP5 that may contribute to its anti-inflammatory activity (Table 1). Identified compound candidates, such as mudanpiosides, paeoniside and suffructicoside, appeared to exhibit strong signal responses in reverse-phase LC-MS in a negative ion mode. To examine the quality of MDP5 amongst batches, we summarized extracted ion chromatograms and the exact intact mass spectra of identified compound candidates to create the MDP5 chemoprofile (Figure 7A and Supplementary Figure S3). The relative signal response was also documented as a signature of MDP5 (Figure 7B).

**TABLE 1:** Identified compound candidates in MDP5.

Identified compound candidates	Formula	Observed m/z	Expected RT (min)	^a^Signal counts	^b^Relative %
(+)-Catechin-7-O-b-glucopyranoside	C22H26O10	449.1454	4.7	3,414,313.2	5.6365
1,2,3,4,6-Penta-O-galloyl-b-D-glucose	C41H32O26	939.1116	4.54	679,457	1.1217
2,5-Dihydroxy-4-methoxyacetophenone	C8H8O3	151.0398	5.27	75,103.6	0.1240
30-Norhederagenin	C29H44O4	455.3165	7.55	760,276.3	1.2551
3-Hydroxy-4-methoxyacetophenone	C9H10O3	165.0555	5.7	186,266.2	0.3075
3-Hydroxy-4-methoxybenzoic acid	C8H8O4	167.0343	4.89	1,301.2	0.0021
4-O-methylbenzoyloxypaeoniflorin	C31H34O13	613.193	5.96	29,111.4	0.0481
4-O-methylgalloyloxypaeoniflorin	C31H34O16	661.1773	4.97	91,537.3	0.1511
4-O-methyloxypaeoniflorin	C24H30O12	509.1652	5.37	84,687.3	0.1398
6-O-Vanillyoxypaeoniflorin	C31H34O15	645.183	5.53	56,701.7	0.0936
Benzoic acid	C7H6O2	121.0293	5.55	8,299.3	0.0137
Catechin	C15H14O6	289.0715	3.91	147,706.6	0.2438
Epicatechin-3-O-gallate	C22H18O10	441.0825	4.48	47,735.8	0.0788
Gallic acid	C7H6O5	169.0138	3.07	1,109,639.4	1.8318
Gallicin	C8H8O5	183.0293	4.23	1,830,077.4	3.0211
Galloyloxypaeoniflorin	C30H32O16	647.1616	4.38	1,749,434.7	2.8880
Hederagenin	C30H48O4	471.3479	7.78	888,149.9	1.4662
Iriflophenone 2-O-b-D-glucopyranoside	C19H20O10	407.0987	3.81	5,503.3	0.0091
Kaempferol	C15H10O6	285.0402	6.1	4,051.4	0.0067
Mudanpioside C	C30H32O13	599.1771	5.52	9,946,953.8	16.4207
Mudanpioside E	C24H30O13	525.1615	4.7	7,908,329.7	13.0553
Mudanpioside H	C30H32O14	615.172	5.05	1,238,160.5	2.0440
Mudanpioside I	C23H28O11	479.1558	4.7	1,615,863.1	2.6675
Mudanpioside J	C31H34O14	629.1877	5.9	14,513,083.7	23.9586
Oxo-acetic acid 2-ethoxy-4-(3-hydroxy-2-oxopropyl) phenyl ester	C13H14O6	265.0713	5.63	1,044.6	0.0017
Oxypaeoniflorin	C23H28O12	495.1507	4.01	2,053,954	3.3907
Paeoniflorigenone	C17H18O6	317.103	4.69	2,514.3	0.0042
Paeoniside B	C30H32O15	631.1667	4.88	7,623,244.3	12.5847
Palbinone	C22H30O4	357.207	6.44	69,722.3	0.1151
p-Hydroxybenzoic acid	C7H6O3	137.0242	5.74	27,439.7	0.0453
Quercetin	C15H10O7	301.0354	5.75	7,791.9	0.0129
Suffrupaeoniflorin A	C36H42O18	761.2307	5.69	10,002.9	0.0165
Suffruticoside D	C27H32O16	611.1621	4.67	3,525,404.6	5.8198
trans-Caffeic acid stearyl ester	C27H44O4	431.3175	8.4	5,041.3	0.0083
Trigalloyl-glucoses	C27H24O18	635.0889	4.22	165,258.7	0.2728
Ursolic acid	C30H48O3	455.3532	8.57	692,413.6	1.1431

a Integration of LC-MS, peak area of extracted ion chromatograms with theoretical m/z ±16.8 ppm from compound compositions.

b The percentage calculated by the relative signal counts to the sum of signal counts from all identified candidates.

Paeoniflorin, gallic acid, and 1,2,3,4,6-penta-*O-*galloyl-β-*D*-glucose (PGG) are reportedly active components with anti-inflammatory functions in MDP (Chen et al., 2020). Among these compounds, gallic acid and PGG are found in the MDP5 fraction (Table 1). Although paeoniflorin was not identified in MDP5, many of its metabolic derivatives, including 4-*O*-methylbenzoyloxypaeoniflorin, 4-*O*-methylgalloyloxypaeoniflorin, 4-*O*-methyloxypaeoniflorin, 6-*O*-vanillyoxypaeoniflorin, galloyloxypaeoniflorin and oxypaeoniflorin, were found in MDP5. Among these derivatives, galloyloxypaeoniflorin (2.89% of the total identified signal) and oxypaeoniflorin (3.90% of the total identified signal) are relatively abundant in MDP5, suggesting that paeoniflorin derivatives may contribute to the anti-inflammatory nature of MDP5 (Table 1).

This investigation identified and elucidated the anti-inflammatory effects of MDP fractions. Among the fractions screened, MDP5 has a dose-dependent immunomodulatory activity on inflammatory signaling and cytokine production. The composition of MDP5 was further identified by mass spectrometry analysis and therapeutic effects of MDP5 were verified in the DSS-induced colitis mouse model.

## Discussion

IBD affects millions of individuals worldwide, in increasingly higher rates (Ng et al., 2018). Since people are commonly diagnosed with IBD at a relatively young age and require life-long medical support, the financial burden resulting from the pricey biologicals, including anti-TNFα, are important issues that must be solved. TCM may be a cost-effective alternative choice for IBD patients. However, concerns surround the therapeutic effects of TCM, because of its complex composition and adverse effects. MDP is a traditional Chinese medicinal material with a long history of use and is recorded in the pharmacopoeias of many countries. MDP is traditionally classified as a “heat-clearing” medicine and is prepared using a decoction of MDP for clinical intake in dosages ranging from 6 to 12 g daily (Committee, 2019). However, the traditional method for MDP preparation may cause diarrhea after long-term use. For practical use in clinics, it is important to have a systematic method for TCM preparation that ensures stable therapeutic efficacy. In this study, we undertook screening to find a therapeutic fraction of MDP that may be applied as a treatment in clinics. This involved a fractionation method combined with the cell-based activity assay and a preclinical study to confirm the therapeutic efficacy of MDP fractions. In the cell-based assay, we also observed increased toxicity when cells were subjected to high concentrations of MDP fractions (Figure 3), which did not necessarily correlate with the anti-inflammatory effect of MDP. The fractionation process can remove the impurities and concentrate the anti-inflammatory components and therefore reduce the cell toxicity or adverse effects of MDP. This study may also overcome the problem of batch and batch differences in TCM preparation by providing a reliable therapeutic TCM fraction chemoprofile for quality control.

In our previous study, the water extract chemoprofile revealed by HPLC-based mass spectrometry demonstrated many important recognized anti-inflammatory compounds, including paeonol, paeoniflorin, gallic acid and PGG (Liu et al., 2018). Interestingly, the water extract, which contains relatively low concentrations of individual compounds, exhibited the strongest anti-inflammatory activity and the highest therapeutic efficacy in the murine colitis model compared with all individual compounds (Chen et al., 2020). This effect may either be the result of a synergistic effect of multiple compounds or is solely due to an as-yet unidentified compound. Therefore, in this study, we performed fractionation of MDP to generate MDP5 with preserved efficacy. Immunoblot analysis of MDP5 revealed the signaling pathway of TLR2 was reduced including IRAK4, TAK1 and the downstream NF-κB signaling, suggesting that all signaling components in the pathway are inhibited (Figure 4). It is likely that the efficacy of MDP5 is due to a synergistic effect of multiple compounds, not exclusively to one unidentified compound, since all of the signaling components are targeted.

Despite the complex composition of MDP5, its chemoprofile revealed that it was mostly composed of known structures that have been reported in MDP (Table 1). It is worth mentioning that compounds with unknown structures may still remain to be identified because of the limitation of LC-MS/MS. Despite the characterization of anti-inflammatory activity for a few MDP5 compounds, such as gallic acid and PGG (Chen et al., 2020), most of the compounds were hitherto uncharacterized, such as galloyloxypaeoniflorin, oxypaeoniflorin, and its derivatives. Thus, it is essential that future research confirms the anti-inflammatory effects of those uncharacterized compounds that may contribute to the therapeutic efficacy of MDP5.

## Data Availability

The original contributions presented in the study are included in the article/Supplementary Material, further inquiries can be directed to the corresponding author.
