# Supplementary material for: Evaluations and Mechanistic Interrogation of Natural Products Isolated From Paeonia suffruticosa for the Treatment of Inflammatory Bowel Disease
Source: Front Pharmacol. 2021 Dec 6;12:696158. doi: 10.3389/fphar.2021.696158 (PMC8686014; doi:10.3389/fphar.2021.696158)
Supplement: Supplementary file 1 [file DataSheet1.PDF]

Supplementary Materials

Evaluations and mechanistic interrogation of natural products isolated from *Paeonia suffruticosa* for the treatment of inflammatory bowel disease

Kun-Chang Wu [1]†, Der-Yen Lee [2]†, Jeh-Ting Hsu [3], Chi-Fang Cheng [4], Joung-Liang Lan [5], Shao-Chih Chiu [4,6,7], Der-Yang Cho [4,6,7,8], and Jye-Lin Hsu [4,6]*

[1] School of Pharmacy, College of Pharmacy, China Medical University, Taichung, Taiwan; kunchangwu@gmail.com
[2] Graduate Institute of Integrated Medicine, China Medical University, Taichung, Taiwan; deryen.lee@mail.cmu.edu.tw
[3] Department of Information Management, Hsing Wu University, New Taipei, Taiwan; 100059@mail.hwu.edu.tw
[4] Graduate Institute of Biomedical Sciences, China Medical University, Taichung, Taiwan; d9359afun@gmail.com (C.-F. C.); t16178@ mail.cmuh.org.tw (S.-C. C.); d5057@mail.cmuh.org.tw (D.-Y. C.)
[5] Division of Rheumatology and Immunology and Department of Internal Medicine, China Medical University Hospital, Taichung, Taiwan; jounglan@me.com
[6] Drug Development Center, China Medical University, Taichung, Taiwan
[7] Translational Cell Therapy Center, Department of Medical Research, Taichung, China Medical University Hospital, Taiwan
8 Department of Neurosurgery, China Medical University Hospital, Taichung, Taiwan

† These authors have contributed equally to this work.
* Correspondence: qiqi0910@gmail.com; Tel.: 886-4-22052121 ext. 7817

Figure S1. Cell toxicity of MDP5 fractions. THP1 cells were treated with different concentrations of MDP5 fractions. After 24 h, THP1 cell viability was determined by the MTS assay. Data are the mean ± S.D. (n = 3–5). **p < 0.01, ***p < 0.001. Data were analyzed using the Student's t-test (two-tailed).

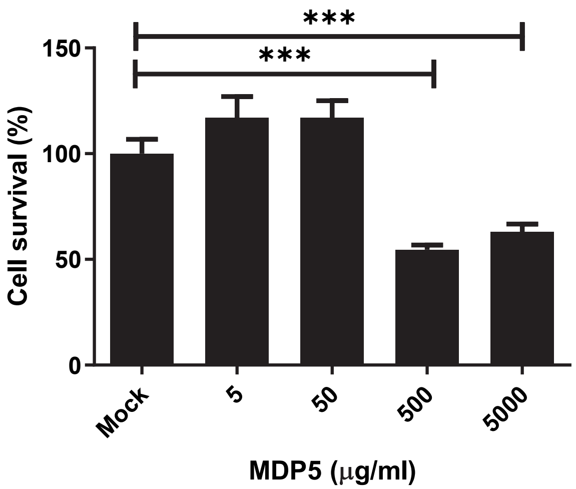

Figure S2. In the treatment group, mice were fed daily with MDP5 (20 mg/kg), starting from day 0 and continuing for 11 days. (A) Body weights and clinical scores were monitored daily. (B) On day 11, blood samples were collected. Indicators of liver function, aspartate aminotransferase (AST/GOT) and alanine aminotransferase (ALT/GPT), and also kidney function, blood urea nitrogen (BUN) and creatinine (CRE), were measured by Hitachi 7080.

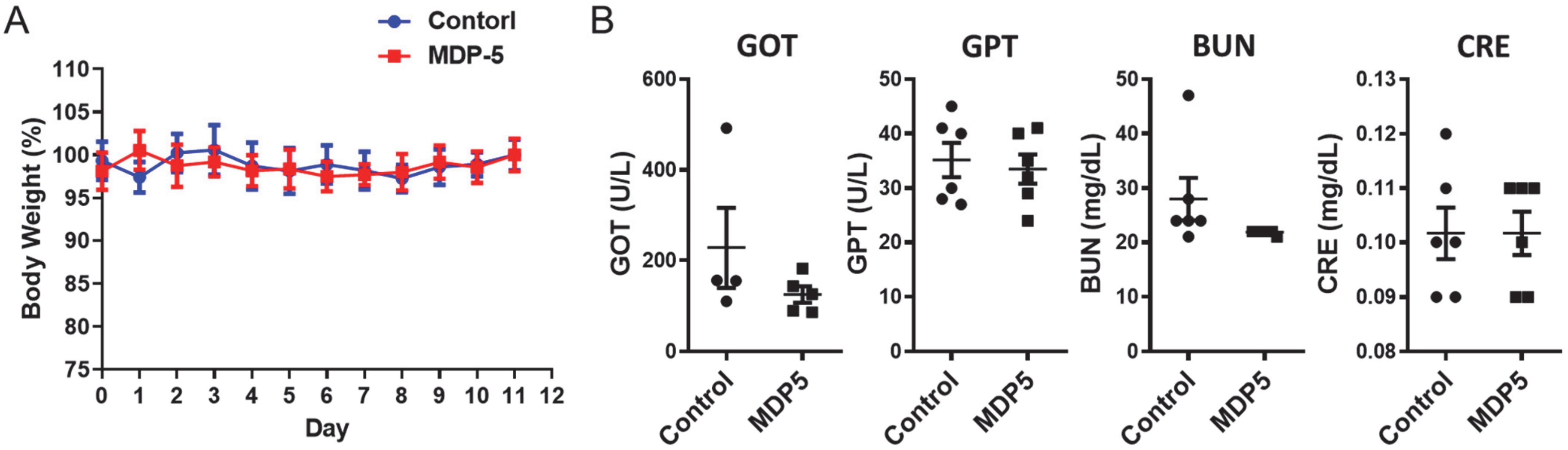

Figure S3. Index compounds in MDP5. (A) The detected mass signals and (B) extracted ion chromatogram (theoretical m/z ±16.8 ppm) for each identified compound candidate.

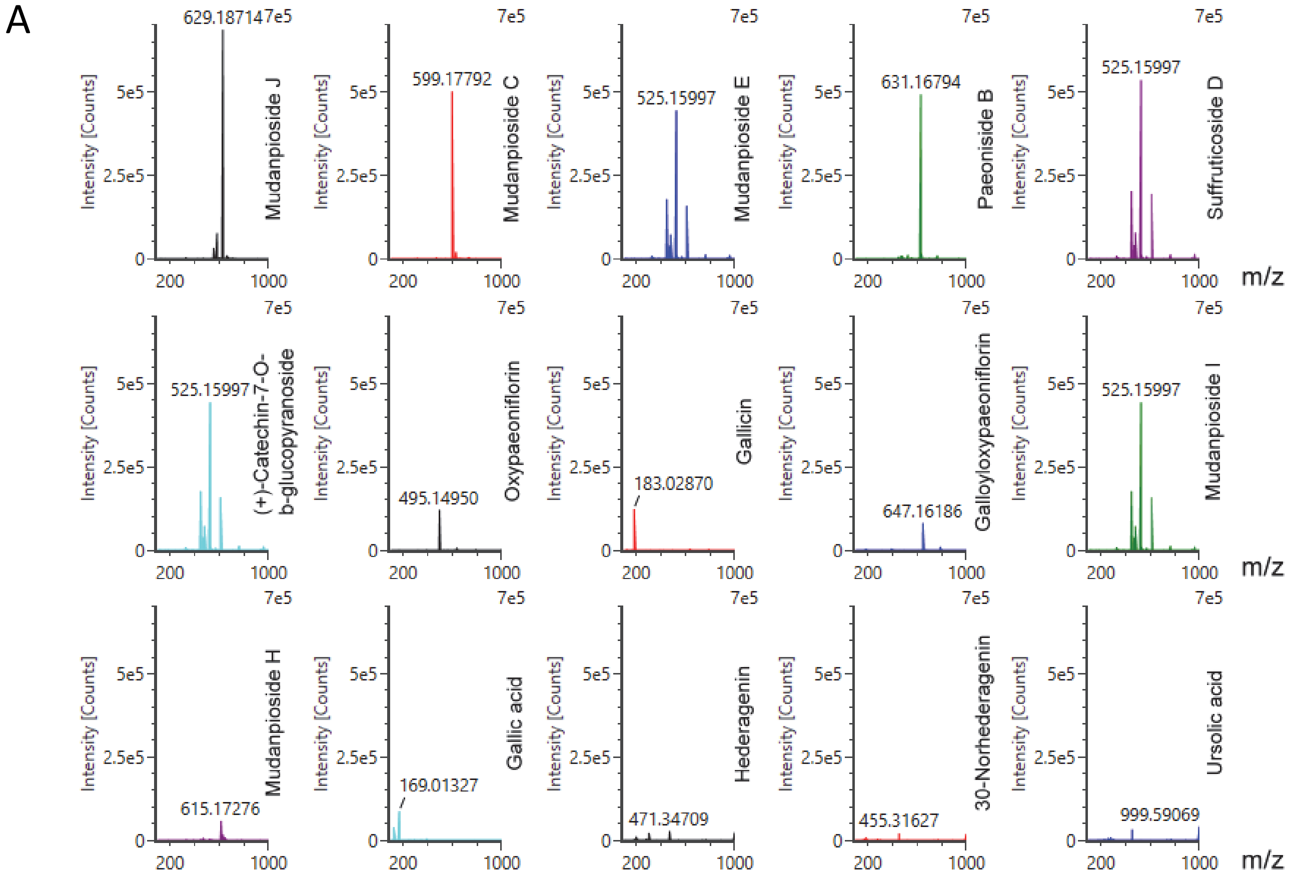

Figure S3

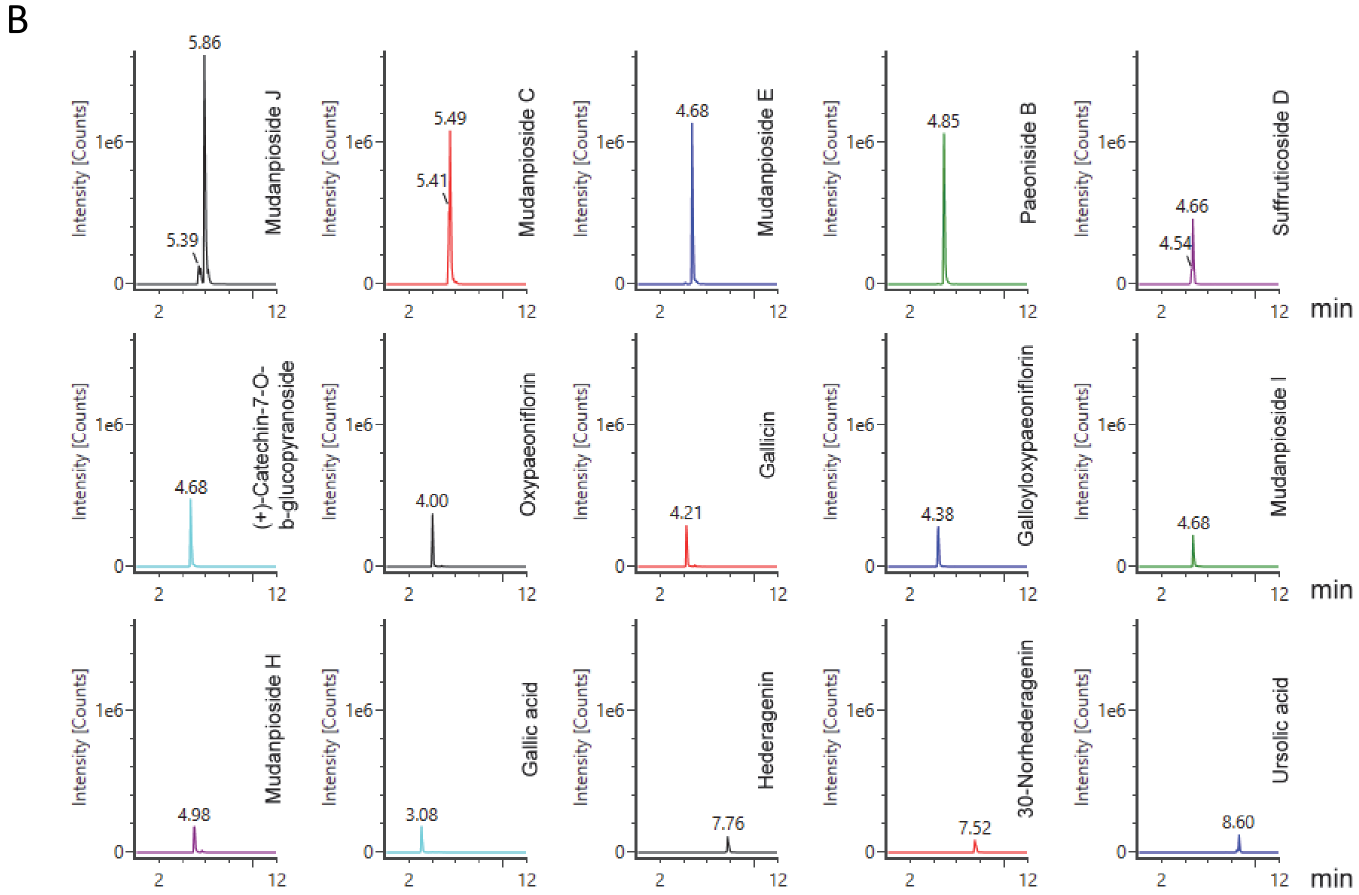
B
Mudanpioside J
5.86
5.39
Mudanpioside C
5.49
5.41
Mudanpioside E
4.68
Paeoniside B
4.85
Suffruticoside D
4.66
4.54
(+)-Catechin-7-O-b-glucopyranoside
4.68
Oxypaeoniflorin
4.00
Gallicin
4.21
Galloyloxypaeoniflorin
4.38
Mudanpioside I
4.68
Mudanpioside H
4.98
Gallic acid
3.08
Hederagenin
7.76
30-Norhederagenin
7.52
Ursolic acid
8.60
Intensity [Counts]
1e6
0
2
12
min
